# A deep learning approach to track Arabidopsis seedlings’ circumnutation from time-lapse videos

**DOI:** 10.1186/s13007-023-00984-5

**Published:** 2023-02-27

**Authors:** Yixiang Mao, Hejian Liu, Yao Wang, Eric D. Brenner

**Affiliations:** 1 Department of Electrical and Computer Engineering, New York University, Brooklyn, NY USA; 2 Biology Department, Pace University, New York, NY USA

**Keywords:** Circumnutation, Plant Movement Tracking, Machine Learning, Deep Learning

## Abstract

**Background:**

Circumnutation (Darwin et al., Sci Rep 10(1):1–13, 2000) is the side-to-side movement common among growing plant appendages but the purpose of circumnutation is not always clear. Accurately tracking and quantifying circumnutation can help researchers to better study its underlying purpose.

**Results:**

In this paper, a deep learning-based model is proposed to track the circumnutating flowering apices in the plant Arabidopsis thaliana from time-lapse videos. By utilizing U-Net to segment the apex, and combining it with the model update mechanism, pre- and post- processing steps, the proposed model significantly improves the tracking time and accuracy over other baseline tracking methods. Additionally, we evaluate the computational complexity of the proposed model and further develop a method to accelerate the inference speed of the model. The fast algorithm can track the apices in real-time on a computer without a dedicated GPU.

**Conclusion:**

We demonstrate that the accuracy of tracking the flowering apices in the plant Arabidopsis thaliana can be improved with our proposed deep learning-based model in terms of both the racking success rate and the tracking error. We also show that the improvement in the tracking accuracy is statistically significant. The time-lapse video dataset of Arabidopsis is also provided which can be used for future studies on Arabidopsis in various takes.

## Introduction

Circumnutation [1] is a term used to describe the back and forth—near elliptical movement of growing plant appendages. Among its known functions, circumnutation is utilized by plant tendrils to locate support for climbing [2] and by plant-parasitic plants for locating prey [3]; but the purpose of circumnutation in most growing plant appendages is still a mystery [4]. Accurately tracking the circumnutation movement over time could be used for comparative analysis during different conditions among and between plant species [1].

Time-lapse videos are an important instrument to characterize plant behaviors including giving a more complete view of circumnutation [5–7]. High-quality images of plant growth and movement can be made at high spatial resolution and low cost by most modern smartphones, which is useful for both laboratory research and classroom activities [6, 8, 9]. We have recently developed *Plant Tracer*, a software that not only tracks, but also quantifies certain parameters of plant movement including speed, distance, and angle of stem curvature [6]. *Plant Tracer* is available for both the cell phone and the computer platform that can be downloaded from the website (https://www.planttracer.com). *Plant Tracer* uses traditional tracking algorithms, i.e. block matching algorithm and Kanade-Lucas-Tomasi (KLT) tracking algorithm. However, these methods lack in robustness on videos taken in a real world context and are not adaptive to scene variability [10]. Recent development in deep learning has revolutionized automated image analysis [11–14], which have shown the near-universal capability to address almost any image processing challenges with high accuracy [15–17]. Neural networks have also benefited plant imaging [18–21], but have not been used for circumnutation studying.

In this work, we create a dataset including the time-lapse videos from the side-view and we develop a deep learning framework to track the flowering stem apex movement and growth. We also develop and publish an executable program using our model, it can be downloaded from our webpage and does not require any coding expertise to set up. The instruction is provided at the end of the paper. We adopt the “U-net” architecture to segment and track the flowering apices in the plant Arabidopsis thaliana from time-lapse videos. The program segments the plant apex in each frame using a trained “U-net” segmentation model with a temporal consistency constraint. Furthermore, the algorithm automatically identifies frames where the segmentation result is inaccurate due to shape changes of the flowering apex and updates the segmentation model using the most recent frames. This segmentation-based tracking method can correct itself if the tracking result of one frame is wrong and is robust to shape changes in flowering apices. Hence, a much longer tracking duration and accuracy can be achieved using our algorithm.

The Related Work section summarizes the related works on object segmentation in video processing, and the previous works on tracking plant movement using video processing technology. The Materials and Methods section introduces our time-lapse video dataset and our deep learning approach for apex detection and tracking in detail. The Results section evaluates the performance of the proposed tracking algorithm and compares our proposed method with 2 widely used plant tracking methods. This section also quantifies the impact of several components of the proposed tracking algorithm. The Discussion section describes the directions to further improve the tracking accuracy and running speed of the proposed tracking algorithm. Finally, the last section concludes this work by listing our major contributions and improvement.

## Related work

Object segmentation is a critical component in many applications, including medical imaging, visual perception, scene understanding, augmented reality, object detection, and image compression, among many other video processing tasks [22–26]. A survey paper [27] summarizes over 100 deep-learning based algorithms for segmentation tasks, and many popular models use the encoder-decoder architecture [28–30]. “U-net” is one of them and was first introduced in 2015 by Ronneberger et al. for segmenting biological microscopy images [31]. Its development in recent years has demonstrated promising performance outside the microscopy image segmentation field [32–35]. However, it has not been adopted to track such plant movement in time-lapse videos.

Tracking seedlings’ movement has been investigated previously. For example, Salma et al. [18] developed deep learning methods to monitor seedlings but they only considered top-view time-lapse videos, and their analysis is designed to monitor the kinetics of early seedling development prior to the emergence of the first true leaf. However, it can be difficult to observe the stem growth in length from the top view. A previous paper [6] uses a block matching method [36] to track the plant (Arabidopsis seedlings) apex from the side view in the time-lapse video. A popular method for object tracking is the Kanade-Lucas-Tomasi (KLT) tracker [37–39] which detects the feature points inside the selected box and calculates the displacement of those points between each frame. This method however has not been adopted for plant tracking in the literature. We compare the proposed deep-learning-based method with the block matching and KTL methods and demonstrate significant improvement in tracking success and accuracy over these baseline methods.

The block-based method and other traditional methods [40, 41] have several limitations [42]: they cannot detect the apex so that users have to manually point to the apex location; they cannot track the flowering apex that changes its shape quickly; also the tracking error accumulates over time and the methods tend to follow the wrong result and cannot correct themselves once the tracking fails in some earlier frames.

To overcome those limitations, we design a detection-based tracking algorithm that adopts the “U-net” architecture to first segment and then track the flowering apices in the plant Arabidopsis thaliana from time-lapse videos. Our algorithm ensures that the detection error does not accumulate over time, because the algorithm re-detects the apex location in the next frame in case the wrong result occurs in one frame. Also, a user does not need to manually indicate the initial apex location since the algorithm automatically detects it. However, the user has an option to draw a box surrounding the apex of interest in the first frame, which could be useful in plants with multiple apices.

## Materials and methods

We first describe our setup to record the time-lapse videos of Arabidopsis seedlings and the time-lapse video dataset. Then, we introduce our deep learning approach for apex detection and tracking, including the core segmentation model, relearning mechanism, and other pre- and post-processing steps.

### Video acquisition

**Fig. 1:**
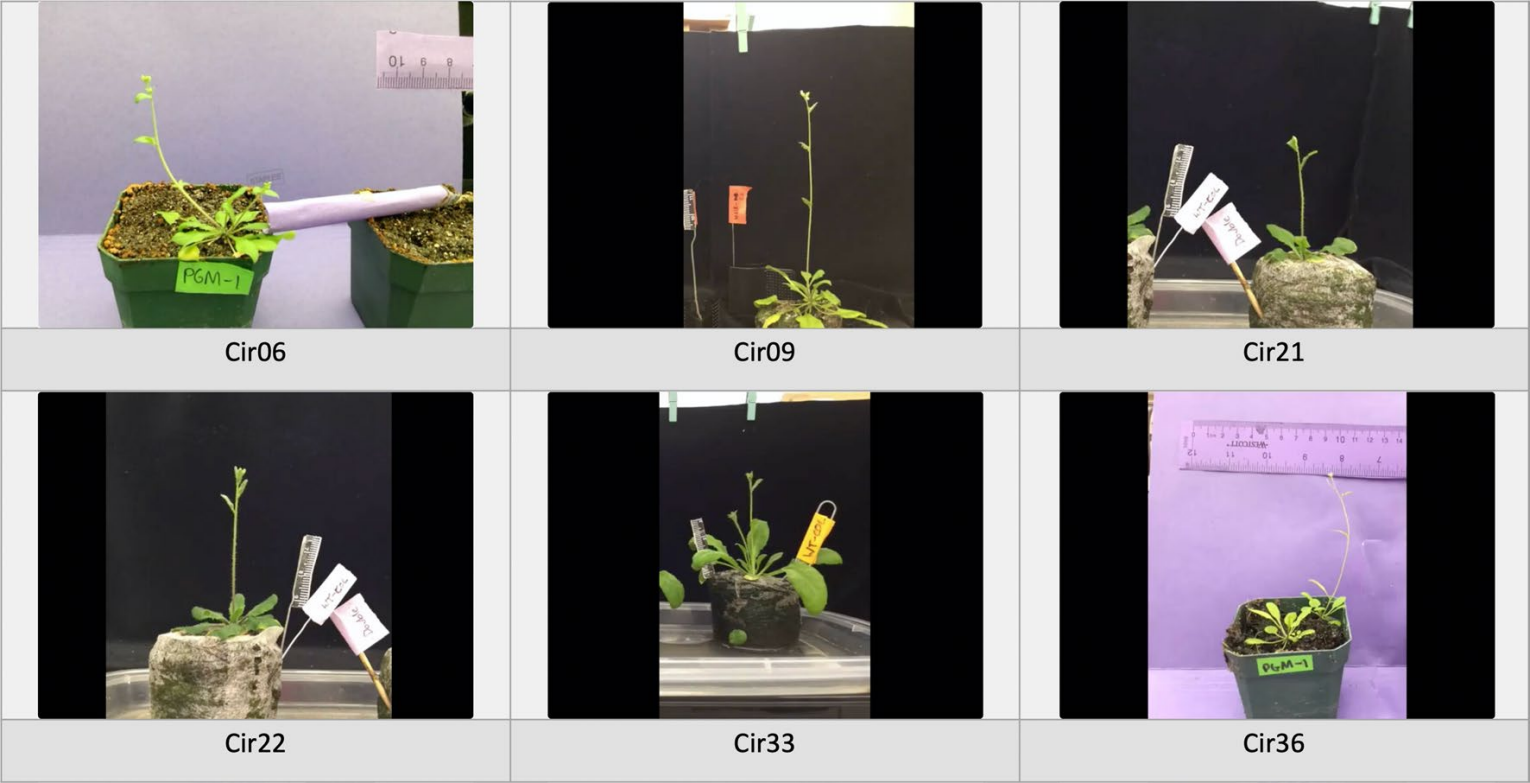
Snapshots of some videos in our time-lapse video dataset of Arabidopsis

The Arabidopsis seedlings were grown according to the method of [5]. Examples of the plant video setup are shown in Fig. 1 and have been previously explained in [5]. It includes a solid color background (typically we use black or purple office folders), a metric ruler (with white lettering and increments set on a black background for best contrast), and identification labels for different plant genotypes and strains. The ruler is used to calibrate the mapping from the pixel counts in the video to the true distance; it is placed in the same focal plane as the plant apices that will be tracked. Labels are placed in close proximity to the plants so that the identity of the plant strain/genotype can be clearly seen in the recordings. An experiment to measure circumnutation on Arabidopsis plant apices was performed. More details on the video capturing setup are described in previous work [6]. For videos with multiple plants in the recording, it was essential that plants are placed at a distance safe enough to avoid possible object occlusion. The Lapse-it app [43] (supported by both iOS and Android devices) is used to create the time-lapse recordings. Typically for Arabidopsis inflorescences, Lapse-it is set to capture one image every two minutes, and then Lapse-it is set to encode the captured images into a time-lapse video at 20 frames per second (fps). Circumnutation movements are recorded for over ten hours to three days.

### Dataset generation

**Fig. 2:**
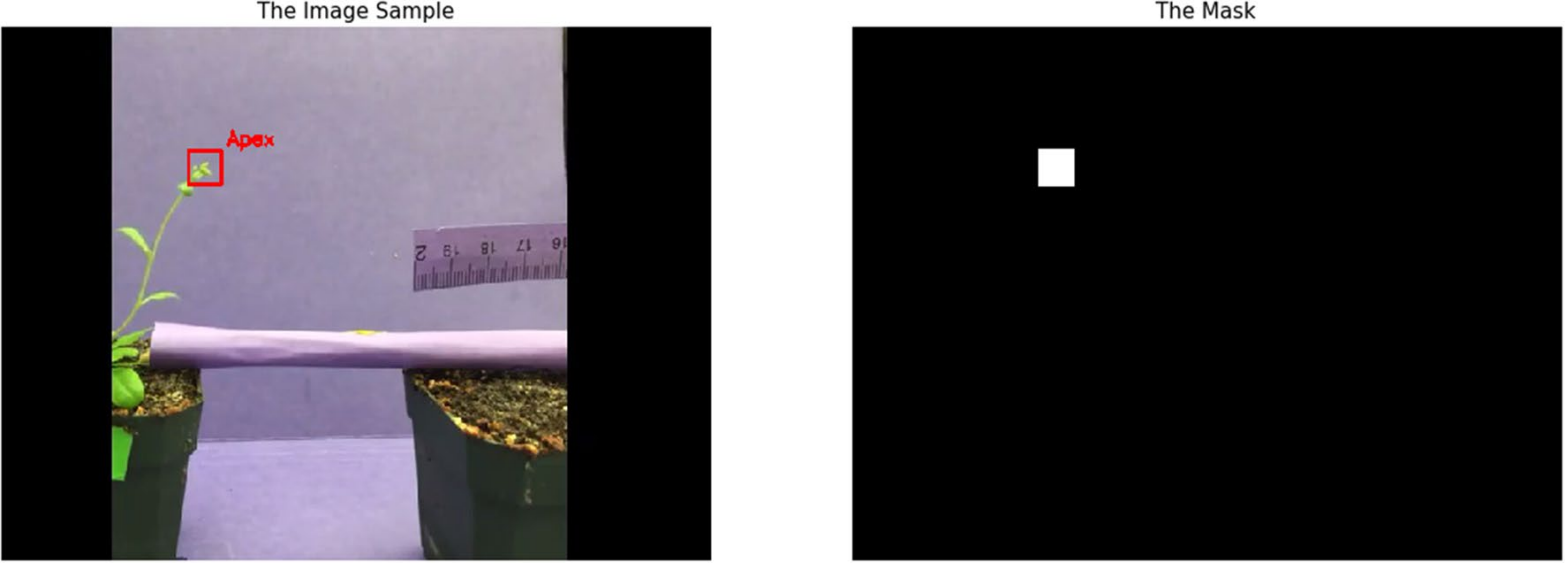
Data generation: **a** the original image ($640\times 480$ pixels), where the red box shows the position of the apex (manually annotated). **b** the ground truth mask image, 1 for pixels inside the box, 0 otherwise, box size is 33*33

We captured 15 videos and annotated the apex location in all frames to generate our dataset. All videos have the same resolution of $640\times 480$ pixels and have 18231 frames in total (corresponding to 607.7 h of plant movement). We split those videos into training, validation, and test datasets, including 10 videos (11056 frames), 2 videos (3471 frames), and 3 videos (3704 frames), respectively. The details of the training videos are listed in Table 1, and the details of the validation and test videos are in Table 2. Some snapshots of the validation and test videos are shown in Fig. 1. To generate the ground truth apex location for our video dataset, we manually annotate a box surrounding the target apex in each frame. Then, a mask map is generated for each frame and the mask is used as the ground truth of the segmentation model. The mask map has a rectangle area (33*33 pixels) corresponding to the annotated box; all pixels inside the rectangle area have a value of 1, and the pixels outside the rectangle area have a value of 0. An example of a frame with its annotated ground truth (mask map) is shown in Fig. 2.

**Table 1:** The information of videos used for training

Video Name	Train_1	Train_2	Train_3	Train_4	Train_5	Train_6	Train_7	Train_8	Train_9	Train_10
Frame number	1427	1778	920	1065	1048	491	1127	1070	1065	1065
Capture time (hour)	47.6	59.3	30.7	35.5	34.9	16.4	37.6	35.7	35.5	35.5
Background color	Black	Black	Black	Purple	Purple	Black	Black	Purple	Purple	Purple

**Table 2:** The information of validation and testing sequences

Video Name	Val_1	Val_2	Test_1	Test_2	Test_3
Frame number	1043	2428	1127	1146	1431
Capture time (hour)	34.8	80.9	37.6	38.2	44.7
Background color	purple	black	black	black	black
Scale (mm/pixel)	0.258	0.276	0.349	0.379	0.276

In this work, we trained the proposed segmentation model independently from other processes using the training dataset. The validation dataset is used to examine the impact of the model’s hyper-parameters and determine the best settings. Then, we report the performance on the test dataset. The Arabidopsis seedling dataset has been made public, including all videos and the manually annotated apex coordinates. The dataset is public and can be used for future studies on Arabidopsis in various takes, e.g., segmentation, recognition, and tracking of Arabidopsis plants. The download link to the dataset is provided at the end of the paper.

### Proposed deep learning approach for apex detection and tracking

**Fig. 3:**
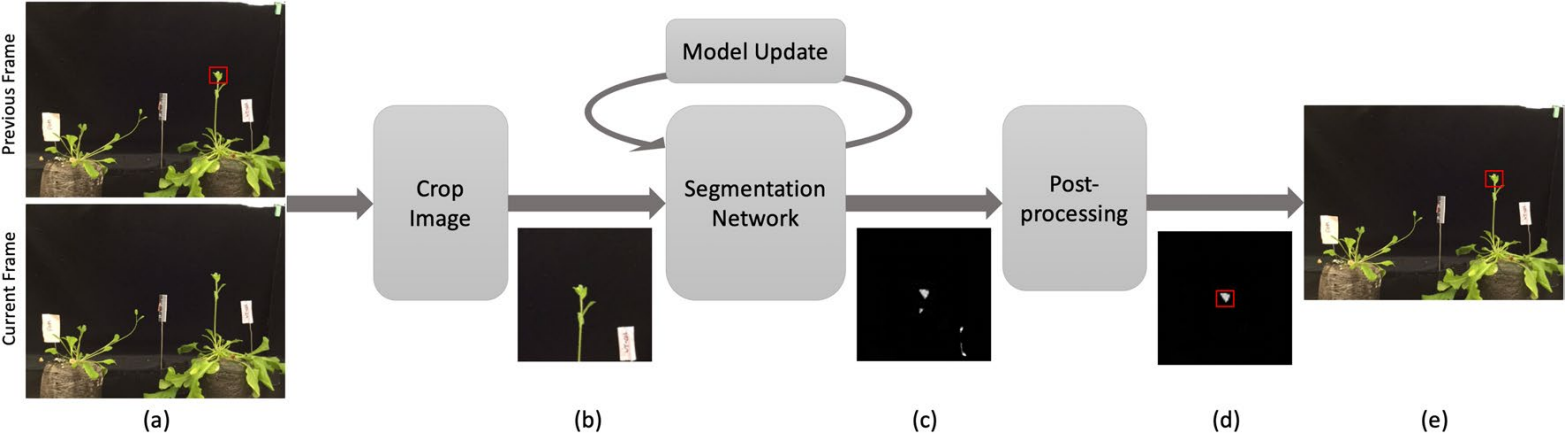
Pipeline of the program: **a** input the previous tracked frame and the current frame. **b** the cropped image. **c** the probability map output by the segmentation model. **d** located apex candidates after removing outliers. **e** the final output image with the tracked apex

The program processes each frame of the input time-lapse video in 3 sequential steps with an additional model update process when necessary. The input frame has $640\times 480$ pixels in 3 channels (red, green, and blue), as shown in Fig. 3a. We first crop the input frame to a square centered at the apex position in the previous frame. The cropped image becomes the input for the segmentation network, as shown in Fig. 3b. Then, the segmentation network outputs a map to indicate the possibility that each point belongs to the apex, as shown in Fig. 3c. Next, post-processing is applied to eliminate outliers. Additionally, when the algorithm suspects the segmentation result of the current frame is grossly wrong, the segmentation network will be updated using the last segmented frame as the ground truth, we call this step *Model Update*. The final output image with the tracked apex is shown in Fig. 3e.

#### The segmentation network

In the following, we first describe the architecture of the proposed segmentation network, and then present the training strategy.

**Fig. 4:**
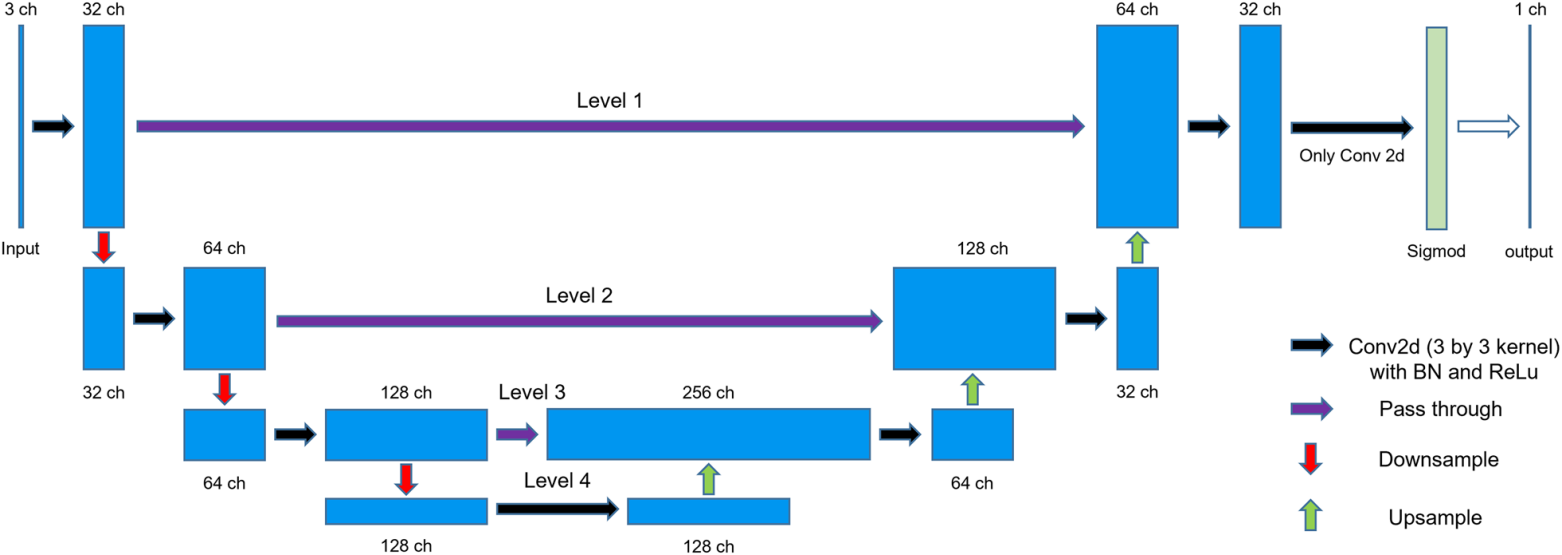
The architecture of the neural network for apex segmentation

Network architecture We adopt a simplified version of the U-net introduced by Ronneberger et al. in 2015 [31]. Figure 4 depicts the overall segmentation network architecture, each blue box corresponds to a multi-channel feature map. The number of channels is denoted on top of the blue box. The network takes the input of an image in 3 color channels (red, green, blue), and outputs a single-channel binary image by thresholding the predicted probability map at the same spatial resolution as the input. Each downsample operation (red arrows in Fig. 4) cuts both the width and height of the feature map by half, and the upsample is the reverse operation (green arrows). The fourth layer is in the lowest resolution ($80\times 60$ pixels for an input of $640\times 480$ pixels). We use a 3*3 kernel size in all 2D convolution layers (black arrows in Fig. 4). We use Rectified Linear Unit (ReLU) [44] as the activation function for all layers except the output layer, where we use the sigmoid non-linearity to generate the output in the range of 0 to 1.

Training the segmentation network The segmentation network is trained separately from the other processes in the pipeline (not an end-to-end training). We use the stochastic gradient descent (SGD) [45] as the optimizer with an initial learning rate of 0.01, and a batch size of 8 images. We choose these hyper-parameters based on the convergence trend and the speed of training the first 3 epochs, each epoch includes 10k+ frames in the validation dataset.

We use a modified Soft Dice coefficient as the loss function. The original Dice coefficient [46, 47] is modified so that it accurately quantifies the intersection of union (IoU), a metric commonly used for assessing image segmentation accuracy. We also apply Laplace smoothing [48] by adding 1 at both numerator and denominator. The final loss function for each training frame can be written as

1 $$\begin{aligned} L=1-\frac{P\cap B+1}{P\cup B+1}=1-\frac{\sum _ip_ib_i+1}{\sum _ip_i+\sum _ib_i-\sum _ip_ib_i+1}, \end{aligned}$$

where *P* denotes the probability map (the output of the segmentation network) and *B* denotes the ground truth mask, $p_i$ and $b_i$ denote the value of *P* and *B* in pixel *i*. Laplace smoothing is used to handle vanishing/exploding gradients when *P* and *B* are both close to zero.

#### Post- processing

**Fig. 5:**
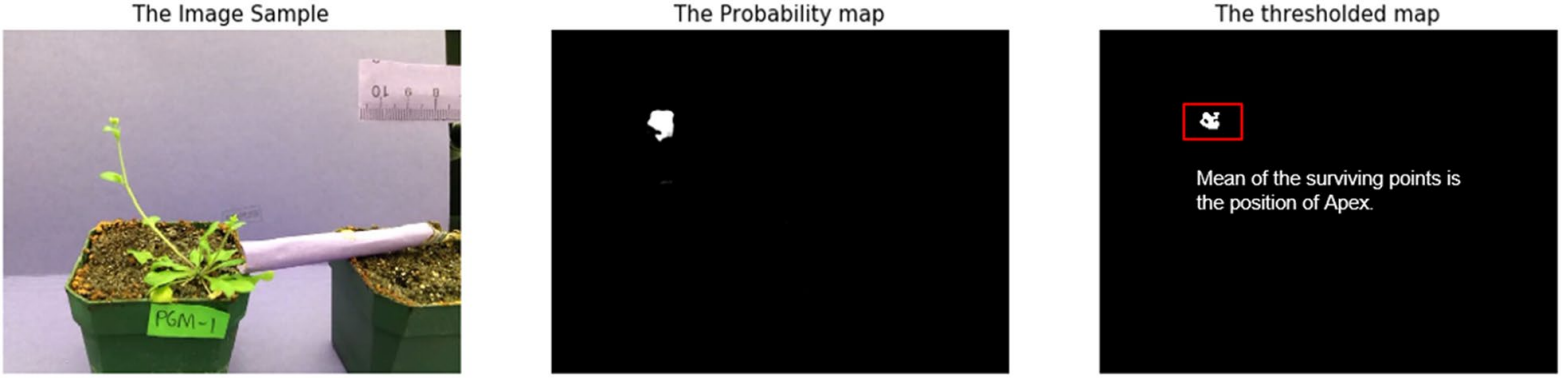
The output probability map and its binary map

**Fig. 6:**
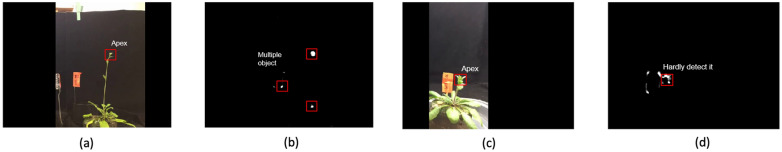
The challenging cases when using only the segmentation network without pre- and post- processing. **a** and **b** show examples of detecting multiple objects. **c** and **d** show examples when the detected center is far away from the actual apex

The segmentation network produces a probability map as shown in Fig. 5. We first threshold the probability map so that pixels with probability $\ge T_m$ are set to 1 (considered as candidate apex pixels) and the remaining pixels are set to 0. We set $T_m = 0.75$ to optimize the performance on the validation dataset. To locate the center of the apex, a naive approach is to simply take the mean coordinates of the candidate apex pixels. However, there are two major problems with this approach: multiple detections and color vanishing. First, the segmentation model sometimes identifies multiple areas to be the possible apex, as shown in Fig. 6b. Secondly, the detected high-probability area sometimes is too large and the center is far away from the actual apex, as shown in Fig. 6d. We noticed that this problem often happens when the apex is large and not monochrome. To solve those problems, we perform several post-processing operations, including search range limitation and outlier removal. The mean coordinates of the remaining pixels are finally used as the apex center.

Set the search range Recognizing that the plant movement within one frame interval (2 min) is limited (less than 20 pixels among all videos from our dataset), the program crops the whole frame into a smaller area covering the detected apex in the previous frame as the segmentation network input to enforce a temporal motion constraint. (The segmentation model’s input for the first frame is still the whole frame.) The area is a rectangle with the size of $2R_1+1$ by $2R_1+1$ pixels centered at the detected apex in the previous frame. This area limitation helps in both eliminating the outliers and speeding up the model inference speed. However, a too-small area will reduce the model inference accuracy, since a smaller local area cannot provide the global plant structure information to the deep segmentation network. Based on the experiment described in the results section below, we set the default $R_1 = 200$ for computers with a powerful GPU, and $R_1 = 50$ for computers without a compatible GPU. Note that $R_1$ does not have to be chosen from the default values; it can also be manually set to any value by users in our executable program, and it should be adjusted based on the actual video resolution and object movement pattern when the model is applied to track plant apices for other types of plants or videos of different resolutions.

Remove the outliers Although the segmentation model only searches for the apex within the search range as mentioned before, the output probability map still occasionally shows multiple high-probability clusters. We first remove all candidate points with a distance of more than $R_2$ pixels from the previous apex center. For those remaining points, the program then removes the candidate pixels which have a large distance to the median of all candidate points. Specifically, the program first determines the medians of horizontal and vertical coordinates respectively of all candidate points, yielding ${{\tilde{x}}}$ and ${{\tilde{y}}}$. Then for each remaining candidate points (*x*, *y*), if $|x-{\tilde{x}}|$ or $|y-{\tilde{y}}|$ is larger than a threshold $R_3$, the point is recognized as an outlier and removed. We test the performance of different settings on the training dataset and set the default as $R_2 = 30$ pixels, and $R_3 = 0.5\cdot R_2$ (or 15 in default). Note that $R_2$ can also be manually set to any value, and it should be adjusted according to the moving speed of the plants and the video resolution.

#### Model update

The pre-trained segmentation model may have difficulty identifying objects that are significantly different from the apices in the training dataset, e.g., an apex from a different species than Arabidopsis. Additionally, some time-lapse videos in our dataset were taken over a period of days, the apex may change in morphology; such that individual flowers may begin to bloom during this long time period, or the lighting condition may change, which may cause the apex to show different color intensity in the video. To accommodate such appearance and color changes, a mechanism called *Model Update* is developed to update the parameters of the pre-trained segmentation model automatically. Our tracking program also allows the user to optionally specify the position of the apex in the first frame to update the pre-trained model, which is important when the program is used for a plant species not included in the training stage.

In each frame, the algorithm generates an initial binary segmentation map using all the previous steps. Then, the algorithm examines the loss value between this initial segmentation map and the segmentation model probabilistic output using the loss function shown in Equation (1). This value is denoted as *suspicion*. If $suspicion>T_{s}$, the segmentation model will be refined with the current frame data only (i.e. using the raw image and the initial segmentation map as the ground truth for this frame). This process will repeat until $suspicion<c\cdot T_{s}$. Once the *Model Update* completes (with $suspicion < c\cdot T_{s}$), the model adapts to the morphology of the apex in the current frame, and this model will be used for subsequent frames until the *Model Update* is triggered again. A too-small *c* can make the model overfit with the feature of this frame and trigger the *Model Update* unnecessarily. We choose $c =0.6$ and $T_s=0.95$ in our system, which provide the best results on our validation dataset. Note that the updated model parameters would only last for one tracking task (one video). When the algorithm processes a new video, the segmentation model will be reset to the pre-trained one.

The pre-trained model parameters should be mostly preserved during the relearning, so the segmentation network still maintains good competence in processing general cases (the apexes seen in the training dataset) after relearning in a specific frame. Therefore, the gradient descent is modified in the relearning state to achieve this goal. At the beginning of a tracking task, the pre-trained parameters are recorded as $W_T$. In each iteration of the relearning, the parameters are updated as:

2 $$\begin{aligned} W_{k+1}=(1-\alpha )W_T+\alpha (W_k-\eta _k \nabla L(W_k)), \end{aligned}$$

where *W* denotes the trainable parameters, *k* denotes the iteration time, $\eta _k$ denotes the learning rate, and $L(\cdot )$ denotes the loss function. $\alpha \in (0,1)$ is a weight balancing the pre-trained parameters and the relearned parameters. After the model parameters are updated for the current frame, they are used as new $W_T$, which will be used in the next possible relearning in the same video. Note that $W_T$ is reset when starting a new video.

In addition to the *suspicion* condition to end the relearning, we also set a time threshold to prevent the *suspicion* from taking too much time to reach $suspicion<c\cdot T_{s}$. Specifically, if the time of relearning exceeds the time threshold, the program terminates the relearning and moves on to process the next frame. In our program, we set this time threshold to 3 s.

## Results

In this section, we first evaluate the performance of the proposed tracking algorithm. Then, we compare it with the widely used Kanade–Lucas–Tomasi (KLT) tracker [37–39] and a modified block matching (BM) tracker used in our previous paper [6]. Next, we quantify the impact of several components of the proposed tracking algorithm (including relearning and thresholds in the post-processing steps) to better understand the trade-off between tracking performance vs. computation time afforded by these components.

### Tracking performance on real time-lapse videos

We evaluate the performance of our program using the metrics of the tracked time and the tracking error. The tracked time is the number of frames the algorithms can track until any “fatal” failure happens. A fatal failure means the program is unable to correct itself and locate the apex in future frames. We report the percentage of frames where the algorithm can successfully track its target apex, which is called this the Tracking Success Rate (TSR). We further calculate the error (pixel distance) between the ground truth coordinate $(x_i,y_i)$ of the apex center and the predicted coordinate $(\hat{x_i},\hat{y_i})$ in each frame before the tracking failure. We use the Euclidean distance defined as $\epsilon _i = \sqrt{(x_i-\hat{x_i})^2+(y_i-\hat{y_i})^2}$, and determine the mean and standard deviation of this error over all successfully tracked frames.

We compare the tracking performance of our algorithm with the two baselines over validation and test videos. The first baseline (the KLT tracker) detects the feature points inside the selected box and calculates the displacement of those points between each frame. We use the publicly available implementation of KLT tracker [49] for this baseline. The second baseline is a modified block matching (BM) tracker used in our previous paper [6]. Table 3 reports the TSR, and the mean and the standard deviation of the errors for the validation and testing videos.

**Table 3:** The performance of the proposed model, the KLT tracker, and the BM tracker. (TSR: Tracking Success Rate)

Video	Proposed method			KLT tracker			BM tracker		
	TSR	Error mean (mm)	Error std (mm)	TSR	Error mean (mm)	Error std (mm)	TSR	Error mean (mm)	Error std (mm)
Val_1	100%	**0.32**	0.19	100%	1.16	0.28	0.0%	N/A	N/A
Val_2	**100%**	1.52	0.72	34.8%	1.84	0.80	70.4%	4.17	0.75
Test_1	100%	**0.91**	0.48	100%	1.13	0.65	100%	2.46	0.64
Test_2	**100%**	0.94	0.53	98.0%	1.01	0.44	42.5%	1.04	0.41
Test_3	100%	**1.43**	0.52	100%	2.10	0.93	25.2%	3.19	0.59
Average	**100%**	**1.02**	0.49	86.6%	1.45	0.62	47.6%	2.72	0.60

The bold values indicate that the corresponding algorithm (or model setting) performs the best in the comparison

As shown in Table 3, while our proposed tracking algorithm can track all videos to the end, the KLT tracker fails to do so on Val_2 and Test_2. For all videos that the KLT can successfully track, the errors of our proposed algorithm are consistently smaller than the errors of the KLT tracker. Meanwhile, the BM tracker only can track the apex in Test_1 to the end, and results in larger mean errors than the proposed algorithm and the KLT tracker for all the videos that it can track at least partially. Note when a tracker fails before the end of a video, the reported mean and standard deviation of its error may not be comparable with other method, because the reported numbers are obtained over different time periods.

To investigate if the reduction in the tracking errors by the proposed algorithm is statistically significant compared to the baselines, we conduct the Wilcoxon signed-rank test [50] on the error difference between the proposed algorithm and each baseline. The differences in tracking errors are determined before either the proposed method or the baseline method fails for each video. Then, we perform the Wilcoxon test on the error differences to determine the p-value, which is the chance that the difference comes from a distribution whose median is zero. Table 4 reports the p-values from the test. All the p-values are substantially lower than 0.05, demonstrating that the proposed algorithm is statistically significantly better over both baselines.

**Table 4:** The p-value of the Wilcoxon signed-rank test on the tracking error difference

p-value	Val_1	Val_2	Test_1	Test_2	Test_3
$\epsilon _{Proposed} - \epsilon _{KLT}$	7.74e-173	1.45e-06	6.79e-38	5.32e-09	8.52e-82
$\epsilon _{Proposed} - \epsilon _{BM}$	N/A	2.57e-191	2.69e-181	2.38e-19	3.01e-37

### Evaluation of different processes in the framework

We quantify and analyze the impact of each component of the proposed tracking algorithm, including the adjustable search range and the *Model Update*.

#### Impact of the searching range $R_1$

It is critical for the segmentation network to achieve real-time inference on users’ personal computers or cell phones. An effective way to speed up the inference is to crop the completed video frame to a smaller image (we call it *search range*) as the model input. In this case, the algorithm only needs to process this cropped region of the whole frame and the processing time can be significantly reduced depending on the cropped image size. However, a smaller cropped image may reduce the model inference accuracy because it is difficult for the model to extract features from an image that is much smaller than training images. In general, a larger search range provides more accurate tracking but the algorithm processing speed is slower.

**Table 5:** Effect of the search range on the tracking error and computation speed

Search range Video	$101\times 101$		$201\times 201$		$401\times 401$	
	Error (mm)	Speed (fps)	Error (mm)	Speed (fps)	Error (mm)	Speed (fps)
Val_1	1.82	**93.6**	2.54	73.9	**0.32**	31.5
Val_2	1.45	**94.1**	**1.40**	73.7	1.52	30.5
Test_1	2.53	**91.3**	1.68	73.3	**0.91**	30.5
Test_2	2.82	**89.4**	1.51	72.6	**0.94**	31.7
Test_3	**1.37**	**92.2**	1.85	74.7	1.43	31.7
Average	2.00	**92.1**	1.79	73.6	**1.02**	31.2

The speed is measured on a Nvidia RTX 2080 Max-Q laptop GPU. When running on an Intel i9-10980HK CPU, on average our program runs at 33fps, 16fps, and 6fps when the search range is set to $101\times 101$, $201\times 201$, and $401\times 401$, respectively.

The bold values indicate that the corresponding algorithm (or model setting) performs the best in the comparison

We evaluate such trade-off between the inference speed and accuracy over 3 different search ranges, e.g., $101\times 101$ pixels, $201\times 201$ pixels, and $401\times 401$ pixels, as shown in Table 5. The results are measured on a laptop with Nvidia RTX 2080 Max-Q GPU. Compared to the smaller search ranges, the search range with $401\times 401$ pixels achieves a significantly lower tracking error (around 50% lower on most videos), because such a large search range is close to the original frame size, and the model is trained to extract features on such scale. However, the model needs more time to process the input with such a large search range. In particular, the model can process the $401\times 401$ input at around 30 frames per second (fps) using the RTX 2080 Max-Q GPU, while it can process the $201\times 201$ input at around 73fps or the $101\times 101$ input at around 90fps.

We also measure the inference speed only using the CPU on our laptop (Intel i9-10980HK). On average, our program runs at 33fps when the search range is $101\times 101$, while running at 16fps and 6fps when the search range is set to $201\times 201$ and $401\times 401$, respectively. In order to reach real-time inference (20fps), our executable program benchmarks the speed of the computer and sets the search range accordingly. For instance, once the executable program is opened, it runs the inference on an example image with the $401\times 401$ resolution. If the inference takes less than $T_1$, our program chooses to use the $401\times 401$ search range to ensure a better tracking result; if the inference takes more than $T_2$ second, our program adapts to the $101\times 101$ search range to reduce the inference time and consequently reduce the user’s waiting time; if the inference time on example images takes the time in between $T_1$ and $T_2$, it chooses to use the $201\times 201$ search range. Based on our experiment, we set $T_1=0.04$ second and $T_1=0.17$ second.

#### Impact of *Model Update*

To verify the impact and performance of the *Model Update* mechanism, we also evaluate our model when the *Model Update* is disabled. Relearning is designed to work especially in situations when the tracking accuracy is low, so we compared the proposed algorithm and the algorithm with disabled *Model Update* when the search range is $101\times 101$ pixels, as shown in Table 6. We discover that even with the relearning enabled, *Model Update* is triggered rarely, typically 1 to 3 times through an entire video sequence. Therefore, it does not really have any consistent impact on the inference time. Compared to the algorithm with relearning disabled, the algorithm with relearning enabled always achieves better results. Specifically, for the videos (Val_1, Test_2), the relearning-disabled model fails to track to the end, while the relearning-enabled model can always track for a longer time before any failure. For other videos, our proposed relearning-enabled model always achieves lower tracking error. Thus, we confirm that our proposed relearning mechanism is effective.

**Table 6:** Effect of the relearning mechanism

Video	Relearning disabled			Proposed, relearning enabled			
	TSR	Error (mm)	Speed (fps)	RT	TSR	Error (mm)	Speed (fps)
Val_1	9.2%	N/A	N/A	1	**100%**	1.82	93.6
Val_2	100%	3.78	92.2	1	100%	**1.45**	**94.1**
Test_1	100%	2.79	**91.9**	1	100%	**2.53**	91.3
Test_2	87.6%	N/A	N/A	1	**100%**	2.82	91.4
Test_3	100%	3.44	**92.4**	1	100%	**1.37**	92.2
Average	79.4%	N/A	N/A	1	**100%**	N/A	N/A

Results obtained with a search range of $101\times 101$ on the GPU. RT (relearning times) indicates the number of times that relearning is triggered within the entire video

The bold values indicate that the corresponding algorithm (or model setting) performs the best in the comparison

## Discussion

Compared to baseline algorithms, the proposed algorithm significantly improves both the tracking success ratio and the tracking accuracy on our dataset, which should correspond to substantial improvement in the overall capability of tracking plant apices. We have made public our annotated video dataset, which includes 15 videos containing a total of 18231 frames, with manually annotated apex coordinates. The download link to the dataset is provided at the end of the paper. This dataset can be used in future studies on Arabidopsis seedlings and can be used for training more advanced neural networks for apex detection or tracking.

There are several directions we are working on to further improve the tracking accuracy and running speed of the tracking algorithm. To remove the interference from the background objects in the video, we can add a “color filter” on the input of the segmentation network to only keep areas with white and green colors, with preliminary attempts showing encouraging results. Additionally, the current program needs to reduce the size of the search range to achieve a tolerable real-time running speed on devices without powerful GPUs, which may lead to lower tracking accuracy. Another potential solution to further reduce the inference time without sacrificing the accuracy is to periodically run the proposed tracking algorithm once every *N* frames, and apply a faster tracker (e.g., KLT) on the remaining frames.

## Conclusion

In this work, we proposed and developed an algorithm to track the movement of Arabidopsis seedling apices in time-lapse videos. We demonstrate that both the success ratio and the accuracy of tracking the flowering apices in the plant Arabidopsis thaliana are substantially improved with our proposed deep learning-based model, and the improvement is statistically significant. By utilizing the deep-learning based object segmentation network that detects the apex location in each frame, together with the proposed relearning mechanism and several pre- and post-processing steps, our algorithm can achieve a 100% tracking success ratio with the smallest tracking error on our dataset, even under challenging scenarios. By introducing *Model Update*, the system can track the changing apex or the apex unseen in the training dataset, without retraining the deep neural network from scratch. Meanwhile, we contribute the circumnutation video dataset of Arabidopsis, which can be used for future studies on Arabidopsis in various tasks.

## Data Availability

Our dataset of circumnutation of Arabidopsis seedlings is public, which includes 15 time-lapse videos containing a total of 18231 frames (corresponding to 607.7 h of circumnutation recording) with manually annotated apex coordinates. All videos have the same resolution of $640\times 480$ pixels. Those videos are separated into training, validation, and test datasets, including 10 videos (11056 frames), 2 videos (3471 frames), and 3 videos (3704 frames), respectively. The details of the training videos are listed in Table 1, the details of validation and test videos are in Table 3. Some snapshots of the validation and test videos are shown in Fig. 1. This dataset can be used in future studies on Arabidopsis seedlings and can be used for training more advanced neural networks for apex detection or tracking. It can be downloaded from: https://drive.google.com/drive/folders/1_ieEnzIJXS5DWnIeE34GrQ-Mz2V0e5Ns?usp=sharing. We develop and publish an executable program using our model. Note that the purpose of this executable program is only to demonstrate the proposed tracking algorithm, and it was only tested on the circumnutation videos provided in the dataset. The current program only works on videos of spatial resolution of $640\times 480$ pixels. To apply the program on videos of other resolutions, the videos should be first resized to $640\times 480$. The program can be downloaded from: https://drive.google.com/drive/folders/1A5bhM96IU_aMGPbF_0lj1Cl9_JS4PkC8?usp=sharing
